# Determinants of anemia status among pregnant women in ethiopia: using 2016 ethiopian demographic and health survey data; application of ordinal logistic regression models

**DOI:** 10.1186/s12884-022-04990-8

**Published:** 2022-08-26

**Authors:** Kassahun Animut, Getasew Berhanu

**Affiliations:** Department of Statistics, College of Natural and Computational Science, Dilla University, Dilla, Ethiopia

**Keywords:** Anemia, Pregnant women, Ordinal logistic regression, Partial proportional odds model, Marginal effect, Ethiopia

## Abstract

**Background:**

Anemia is a serious public health problem that occurs when the blood contains fewer red blood cells than normal. In Ethiopia, the prevalence of anemia in pregnancy increased between 2005 and 2016. The aim of this study was to determine what factors influence the anemia status of pregnant women in Ethiopia.

**Methods:**

Anemia status in a sample of 1053 pregnant women was studied using data from Ethiopia's Demographic and Health Survey 2016. Percentages and graphs were used to show the prevalence of anemia. The marginal probability effect was used to determine the contribution of each explanatory variable category to a single response category of anemia level. Ordinal logistic regression models were constructed, and the best-fitting model was selected to reveal significant anemia status variables.

**Results:**

The prevalence of anemia in pregnant women was found to be 37.51% (3.04% severe, 17.28% moderate, and 17.1% mild anemic). The fitted partial proportional odds model revealed that anemia status of pregnant women was significantly associated with region afar (OR = 0.45; CI: 0.21–0.96), antenatal care visits above 4 (OR = 1.58; CI: 1.03–2.43), parity between 1–2 (OR = 0.47;CI: 0.26–0.85), iron taking (OR = 3.68;CI: 2.41–5.64), and higher education (OR = 4.75;CI: 2.29–9.85).

**Conclusions:**

Anemia among pregnant women has been identified as a moderate public health issue in Ethiopia. The study revealed that the prevalence of anemia varied among regions which the highest (65.9%) and the lowest (9%) being from Somali and Addis Ababa, respectively. As a result, it is argued that treatments target iron consumption, maternal education, antenatal visits, and mothers' access to health care.

## Introduction

Anemia is a worldwide public health issue that affects about 1.62 billion people. Heavy menstrual bleeding and parasite infections such as malaria and HIV are the most common causes of anemia during pregnancy, as they lower hemoglobin levels in the blood [1].

Prenatal anemia is most prevalent in developing countries and accounts for 95.7% of the global burden. Anemia is one of the most critical public health problems in the world. It affects 41.8% of pregnant women worldwide, with rates ranging from 5.7% in the United States to 75% in the Gambia. [2]. In Africa, nearly half of all anemia cases (46.3%) occurred during pregnancy [3]. Anemia lowers hemoglobin levels, which promotes alterations in placental angiogenesis, restricting oxygen supply to the fetus and, as a result, causing intrauterine growth restriction and low birth weight [4].

Anemia, defined as a hemoglobin concentration in the blood of less than 110 g/L, is the second most common cause of disability in the biosphere. Clinical assessment (conjunctival pallor testing) is a common method of diagnosing anemia, but it is imprecise. The African National Congress uses the healthcare incident command system (HICs) to perform a comprehensive blood count and assess the blood hemoglobin level (ANC). However, incorporating this and other established tests in to rural LMIC settings may be prohibitively expensive, difficult to administer, or impractical [5].

In underdeveloped nations, such as Ethiopia, anemia is a major and widespread public health issue. During pregnancy, it causes a variety of issues and difficulties for both the fetus and the mother. According to an Ethiopian demographic and health survey study, one-fifth of reproductive-aged women (15–49) are anemic, with severe, moderate, and mild anemia accounting for 1%, 3%, and 13%, respectively [6, 7]. According to the Ethiopian Demographic and Health Survey [8] report, 24% of women of reproductive age were estimated to be anemic and 29% of pregnant or breastfeeding women were anemic. Among women who had a live birth five years prior to the survey, the prevalence of anemia decreased from 20% in 2005 to 13% in 2011, and the data for 2011 revealed a much broader gap in the prevalence of anemia between pregnant (29.9%) and non-pregnant women (10.8%) [9]**.**

Although several studies have been conducted in Ethiopia, they used a limited number of variables and small sample sizes without indicating the severity of anemia status in pregnant women, and these studies did not consider the impact of each explanatory variable on a single anemia status category. Therefore, the current study was conducted at the national level using 2016 EDHS data to determine the anemia status of pregnant women and identify relevant risk factors.

## Methods

### Data source

The data for the analysis came from 2016 Ethiopian Demographic and Health Survey (EDHS). It is the fourth comprehensive and nationally representative, cross-sectional, population and health survey conducted by the Central Statistical Agency in collaboration with the Federal Ministry of Health (FMoH) and the Ethiopian Public Health Institute (EPHI) with technical assistance from ICF International, and financial as well as technical support from development partners. The 2016 EDHS sample was stratified into urban and rural areas and then selected in two stages.

A total of 645 enumeration areas (EAs) with an average of 181 households were chosen in the first stage, with probability proportional to EA size (202 of them were from urban areas, while 443 were from rural areas). In the second stage, systematic sampling was used to choose 28 households per EA. For the survey, a total of 17,067 households were occupied. A total of 16,650 women were successfully questioned, resulting in a 98 percent response rate. A total of 15,683 women were chosen for the sample from Ethiopia's nine regions and two city administrations, of whom 1,122 were pregnant and 1,053 were successfully questioned [10].

### Data extraction method

After receiving approval from the EDHS program, the 2016 EDHS data were obtained from the DHS program website (http://www.dhsprogram.com). Based on the existing literature, data cleaning, extraction, variable selection, and recoding of the classification of some categorical variables were completed. Sampling weights were used to account for unequal selection probability between strata.

### Inclusion–exclusion criteria

All pregnant women with known hemoglobin levels were included in this study, while women with unknown hemoglobin levels were excluded.

### Variables of the study

#### Dependent variable

The outcome variable was the anemia status of pregnant women aged from 15 to 49. It was determined based on hemoglobin concentrations in the blood. Anemia was defined as the occurrence of hemoglobin levels less than 11 g/dL. It was further categorized in to severe, moderate, mild and not anemic with hemoglobin ranges < 7.0 g/dl, 7.0—9.9 g/dl, 10.0—10.9 g/dl, and ≥ 11.0 g/dl respectively [10, 11].

According to WHO, the prevalence of anemia should be less than 5% and is defined as a mild public health problem at a prevalence of 5% to 19.9%, a moderate problem at a prevalence of 20% to 39.9%, and a severe problem at a prevalence of 40.0% or more [12].

### Explanatory variables

The selections of explanatory variables were theoretically driven that draw support from prior research with regard to factors affecting pregnant women’s hemoglobin levels. Previous studies have been referenced in creating categories for naturally continuous and discrete variables [13–16] (Table 1).

### Statistical analysis

We examined the data for completeness and consistency once it was extracted, and then we completed the preliminary analysis. Descriptive and inferential statistics were used to analyze the data. Different tools, such as frequency distributions, percentages, and graphs, were utilized in descriptive statistics to demonstrate the anemia status of pregnant women.

To determine the relationship between each explanatory variable and the outcome variable, a Chi-square test was used (anemia status).

In the final multivariable logistic regression analysis, factors having a p-value less than 0.15 in the bivariate analysis were included. The variance inflation factors test (VIF < 10) was used to check for multi-co-linearity of the explanatory variables, and no co-linearity was observed between the candidate variables (all the candidate variables had a VIF value of less than 3). The factors of anemia were discovered using the ordinal logistic regression approach. Variables with p-values less than 0.05 were judged to have a statistically significant association with anemia status in the final model. The strength of the link was assessed using an odds ratio with a 95% confidence interval. Data were analyzed using SPSS version 20 and STATA version 15.

### Ordinal logistic regression model

Logistic regression is the basic and popular modeling approach when the dependent variable is dichotomous or polytomous. When the dependent variable has more than two categories, it may be ordered or unordered. Ordinal logistic regression models are used to model the relationship between independent variables and an ordinal response variable when the response variable category has a natural ordering [17]. The proportional odds model estimates the odds of being at or below a particular level of the response variable. It considers the probability of that event and all events before it. If the proportional odds assumption, i.e., the relationship between the independent variables and the dependent variable, does not change as the dependent variable’s categories is not met, then other different ordinal models are used to identify important explanatory variables.

When the proportional odds assumption is met for some but not all explanatory variables, the partial proportional odds model (PPOM) is used, whereas the generalized ordered logit model (GOLM) is used when the proportionality constant can be completely or partially relaxed for the set of explanatory variables [18]. The continuation ratio logistic model (CRM) compares the probability of response to a given category with the probability of higher response. The construction of adjacent-categories logit recognizes the ordering of response variable categories and determines the logits for all pairs of categories [19].

### Parameter estimation

STATA was used to fit all of the above models to the data set (version 15). The variables were chosen with care and from a survey of the literature. The "ologit" command was used to fit the proportional odds model, and then the "Brant" test was used to evaluate the parallel line assumption. For ordinal logistic regression, the model parameters are estimated by the maximum likelihood estimation (MLE) techniques. In general, the method of maximum likelihood produces values of the unknown parameters that best match the predicted and observed probability values. Therefore, it usually uses a very effective and well known Fisher scoring algorithm to obtain ML estimates [20].

### Model selection

In the case of logistic regression, the model selection criteria based on their results, reasonableness, and fit as measured, will be taken as AIC/ BIC. The log-likelihood value of the models is used to compare the ordinal logistic model, i.e., the model with a higher log-likelihood is considered as better fitted. Akaike Information Criterion (AIC) and Baye’s Information Criterion (BIC) are used to compare models, and the model with the smallest absolute AIC and BIC statistic is considered the best model [21].

### Test of overall model fit

The overall model fit in ordinal logistic regression is based on the change in minus2 log-likelihood when the variables are added to a model that contains only the intercept. McFadden's pseudo R-squared statistic was used to compute based on the log likelihood for the model with predictors compared to the log-likelihood for the model without predictors [22], and the significance of individual explanatory variables in the model was checked by using the Wald test. The Pearson and deviance goodness-of-fit test was used to measure the goodness of fit for the model.

### Marginal effects

The average marginal probability effects of predictors on a single level of the response variable are not achievable in ordinal logistic regression. For categorical independent variables, marginal effects are easier to understand and utilize than marginal effects for continuous variables. After adjusting for the other factors in the model, the ME for categorical variables shows how P(Y) varies as the categorical variable moves from one to the other. It's a typical manner of responding to the question, "What effect does the predictor have on the likelihood of the event occurring?" [23].

The average marginal effect (AME) is a measure of the overall effect of the predictors that is used to assess the sorts of associations and magnitudes between explanatory variable levels and response probability levels [24, 25]. The means are just one of many sets of values that could be utilized, and none of them would have sounded problematic to a real person [26].

## Results

### Socio-demographic and other characteristics of study participants

**Fig. 1:**
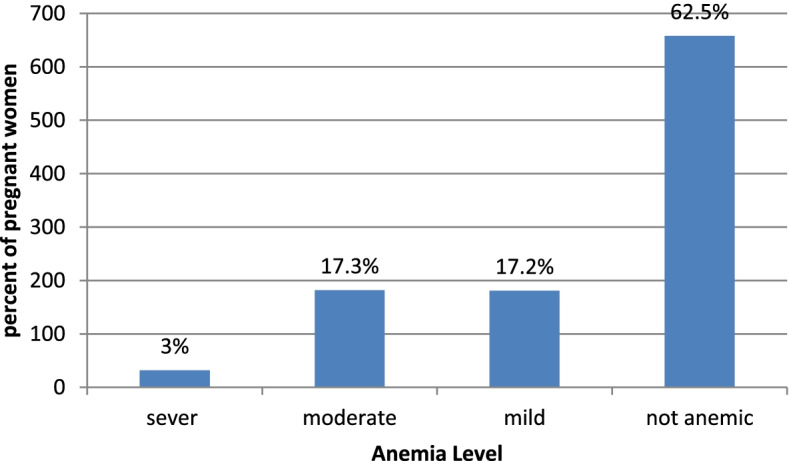
Anemia status among pregnant women’s, EDHS 2016 (*n* = 1053)

This research was based on EDHS 2016 data. A total sample of 1,053 pregnant women at the reproductive age (15–49) was included in this study from those 32 (3.04%) were severely anemic, 214(20.32%) were severe or moderate anemic and 395 (37.51%) were severe, moderate or mild anemic while among all pregnant women 658(62.49%) were non-anemic (Fig. 1).

Table 1 shows the distribution of anemia status among pregnant women based on demographic, socioeconomic, and other factors. A total of, 1053 pregnant women were sampled, with 705 (66.9%) from rural areas and the rest from urban areas. Severe, moderate, mild, and non-anemia were found in 4.4%, 21.42%, 20.71%, and 53.48% of pregnant women from rural areas, respectively. In urban regions, about 80.75% of pregnant women were anemic, while in rural areas, about 53.48% were.

**Table 1:** Socio-demographic and other characteristics of pregnant Women’s anemia status, EDHS 2016 (*n* = *1053*)

Anemia status					
Variables	Severe Count (%)	Moderate Count (%)	Mild Count (%)	Non anemic Count (%)	Total
Age groups					
15–24	14(3.64)	64(16.62)	62(16.10)	245(63.64)	385
25–34	12(2.40)	92(18.12)	84(16.7)	315(62.62)	503
35–39	4(3.45)	17(14.66)	26(22.41)	69(59.48)	116
Above 40	2(4.08)	9(18.37)	9(18.37)	29(59.18)	49
Residence					
Urban	1(0.29)	31(8.91)	35(10.06)	281(80.75)	348
Rural	31(4.40)	151(21.42)	146(20.71)	377(53.48)	705
Educ. Level					
No education	25(5.06)	124(25.10)	88(17.81)	257(52.02)	494
Primary	7(3.80)	42(22.83)	56(30.43)	79(42.93)	184
Secondary	0(0.00)	8(3.52)	30(13.22)	189(83.26)	227
Higher	0(0.00)	8(5.41)	7(4.73)	133(89.86)	148
Wealth index					
poorest	24(6.33)	94(24.8)	72(19.00)	189(49.87)	379
Poorer	3(1.68)	34(18.99)	25(13.97)	117(65.36)	179
Middle	1(0.71)	17(12.06)	23(16.31)	100(70.92)	141
Richer	2(1.54)	15(11.54)	24(18.46)	89(68.46)	130
Richest	2(0.89)	22(9.82)	37(16.52)	163(72.77)	224
Antenatal visit					
No /don’t know	23(5.09)	106(23.45)	73(16.15)	250(55.31)	452
1–3	8(2.50)	48(15.00)	64(20.00)	200(62.5)	320
Above 4	1(0.36)	28(9.96)	44(15.66)	208(74.02)	281
Religion					
Orthodox	1(0.36)	20(7.22)	37(13.36)	219(79.06)	277
Muslim	28(4.84)	140(24.22)	111(19.20)	299(51.73)	578
Others	3(1.52)	22(11.11)	33(16.67)	140(70.71)	198
Parity					
No child	1(0.38)	11(4.23)	13(5.00)	235(90.38)	260
1–2	3(1.06)	26(9.19)	44(15.55)	210(74.20)	283
3–5	6(2.29)	49(18.70)	42(16.03)	165(62.98)	262
Above 6	22(8.87)	96(38.71)	82(33.06)	48(19.35)	248
Birth in 5 years					
No birth	1(0.29)	10(2.87)	21(6.02)	317(90.83)	349
One birth	5(1.32)	54(14.25)	71(18.73)	249(65.70)	379
Above 2 birth	26(8.00)	118(36.31)	89(27.38)	92(28.31)	325
Smoking status					
No	32(3.08)	179(17.21)	179(17.21)	650(62.50)	1040
Yes	0(0.00)	3(23.08)	2(15.38)	8(61.54)	13
Occupation					
Not working	28(4.40)	121(19.00)	115(18.05)	373(58.56)	637
Agricultural	1(0.51)	26(13.33)	33(16.92)	135(69.23)	195
Non agricultural	3(1.36)	35(15.84)	33(14.93)	150(67.87)	221
Visithealthfac12mo					
No /don’t know	22(4.64)	92(19.41)	76(16.03)	284(59.92)	474
Yes	10(1.73)	90(15.54)	105(18.13)	374(64.59)	579
Marital status					
Unmarried	3(7.32)	4(9.76)	5(12.20)	29(70.73)	41
Married	29(2.87)	178(17.59)	176(17.39)	629(62.15)	1012
Iron taking status					
No /don’t know	31(4.37)	168(23.66)	146(20.56)	365(51.41)	710
Yes	1(0.29)	14(4.08)	32(10.20)	293(85.42)	343

The percentage of pregnant women with severe and moderate anemia was higher in the age group over 40, but the proportion of pregnant women without anemia was higher in the age group 15–24. Severe, moderate, and mild anemia was found in higher proportions in pregnant women from the poorest households (Table 1).

The proportions of severe and moderate anemia status were decreased as the number of antenatal care visits of pregnant women increased while the proportion of non-anemic increased, with the number of antenatal care visits. Pregnant women who had no child developed a lower proportion of severe, moderate, and mild anemia levels than pregnant women who had one/ more children. As the number of births within the last five years for pregnant women increased, the proportion of non-anemia decreased from 90.83% in no birth to 28.31% in the above two births.

The percentages of severe, moderate, and mild anemia were higher in pregnant women who didn’t take iron but had a lower proportion of not anemic than pregnant women who took iron pills (Table 2).

### Ordinal logistic regression analysis

A chi-square test of association was performed before the ordinal logistic regression model was run, and then significance explanatory factors were put into the model at a 15% level of significance. Because the parallel line assumption was violated by the Brant test (chi-square = 100.55, *p*-value = 0.015), the proportional odds model was ruled out, and the data was fitted with partial proportional, generalized ordered logit, continuation ratio, and adjacent category logit models. Finally, a model comparison was performed (Table 2). The best match, according to AIC and BIC values, is PPOM, which has the smallest AIC and BIC (Table 2).

**Table 2:** AIC, BIC and Pseudo R2 for all five ordinal models

Model	Obs	DF	AIC	BIC	Pseudo R^2^
POM PPM GOM CRM ACM	1,053 1,053 1,053 1,053 1,053	39 51 110 63 84	1462.679 1426.994 1479.272 1562.733 1478.135	1656.095 1619.924 2024.805 1875.176 1894.725	0.3465 0.3746 0.4057 0.3432 0.3817

*Key*: Prob > chi2 = 0.0000 for all models

The Pearson goodness of fit test supported that the PPOM model was well-fitted to the data (chi-square = 751.99, *p*-value = 1.00). Thus, PPOM was used to identify significant determinants of hemoglobin level and parameter estimates of this model are presented and interpreted for the significant predictors (at a 5% significance level).

### Results of Partial Proportional Odds Model (PPOM)

Table 3 shows three contrasting result panels. The contrasts are severe versus moderate, mild and not anemic, severe and moderate versus mild and not anemic, and severe, moderate and mild versus not anemic.

**Table 3:** Revealed results of the parameter estimates for the PPOM model, EDHS 2016 (*n* = *1053*)

Predictors		Severe versus moderate, mild and not anemic				Sever and Moderate versus mild and not anemic				Sever, moderate and Mild versus not anemic			
		Coef	p-value	OR	95%CI	Coef	P-value	OR	95%CI	Coef	P-value	OR	95%CI
Region	Afar	-0.805	0.004	0.447	0.208–0.961	-0.805	0.004	0.447	0.208–0.961	-0.805	0.004	0.447	0.208–0.961
	Amhara	0.3003	0.526	1.351	0.534–3.415	0.3003	0.526	1.350	0.534- 3.414	0.3003	0.526	1.350	0.534- 3.415
	Oromia	-1.4192	0.030	0.242	0.067- 0.875	0.2764	0.58	1.318	0.496- 3.508	-0.0231	0.961	0.977	0.387 -2.465
	Somali	-1.5677	0.001	0.209	0.080- 0.544	-1.5677	0.001	0.209	0.080–0.543	-1.5677	0.001	0.209	0.080- 0.544
	Benishangul	-0.2498	0.611	0.779	0.297- 2.040	-0.2498	0.611	0.779	0.297- 2.04	-0.2498	0.611	0.779	0.297- 2.040
	SNNP	0.3715	0.434	1.450	0.572- 3.674	0.3715	0.434	1.449	0.572–3.674	0.3715	0.434	1.449	0.572- 3.674
	Gambela	-0.3953	0.466	0.673	0.233- 1.949	-0.3953	0.466	0.673	0.233- 1.949	-0.3953	0.466	0.673	0.233–1.949
	Harari	-0.7337	0.158	0.480	0.174- 1.329	-0.7337	0.158	0.480	0.174–1.329	-0.7337	0.158	0.480	0.174–1.33
	Addis Ababa	-0.6742	0.268	0.510	0.155- 1.679	-0.6742	0.268	0.510	0.155- 1.679	-0.6742	0.268	0.510	0.155–1.679
	Dire Dawa	-1.4482	0.011	0.235	0.077–0.713	-1.4482	0.011	0.235	0.077- 0.713	-1.4482	0.011	0.235	0.077–0.713
Educational Level (Ref:no education)	Primary	-0.1539	0.746	0.857	0.338 -2.174	-0.1566	0.531	0.855	0.524- 1.40	-1.0698	0.000	0.343	0.208–0.567
	Secondary	0.594	0.243	1.81	0.668 -4.898	1.901	0.000	6.692	2.85–15.69	0.6843	0.013	1.982	1.15–3.407
	Higher	1.56	0.000	4.746	2.288- 9.847	1.56	0.000	4.75	2.28- 9.847	1.5574	0.000	4.746	2.288- 9.847
Iron take(ref: no)	Yes	1.304	0.000	3.685	2.407- 5.642	1.304	0.000	3.685	2.406- 5.64	1.304	0.000	3.685	2.407- 5.64
Residence (ref: urban)	Rural	-2.401	0.021	0.091	0.012–0.701	-0.9105	0.003	0.402	0.220–0.736	-1.5574	0.000	0.211	0.121–0.368
Parity (TNCEB) (Ref: no)	1–2	-0.7459	0.013	0.474	0.264- 0.852	-0.7459	0.013	0.474	0.264–0.852	-0.7459	0.013	0.474	0.264–0.852
	3–5	-1.1764	0.000	0.308	0.172- 0.553	-1.1764	0.000	0.308	0.172–0.553	-1.1764	0.000	0.308	0.172–0 .553
	Above 6	-1.9852	0.000	0.137	0.055–0.345	-2.1059	0.000	0.122	0.066–0.225	-3.2486	0.000	0.039	0.020–0.074
Age group (Ref:15–24)	25–34	0.1075	0.541	1.113	0.789–1.57	0.1075	0.541	1.113	0.789–1.57	0.1075	0.541	1.113	0.789–1.57
	35–39	-0.4709	0.080	0.624	0.368–1.058	-0.4709	0.080	0.624	0.368–1.058	-0.4709	0.080	0.624	0.368–1.058
	Above 40	-0.0146	0.968	0.986	0.479–2.026	-0.0146	0.968	0.986	0.479–2.026	-0.0146	0.968	0.986	0.479–2.026
Births in last5 years Ref:No birth	1 Birth	-1.523	0.000	0.218	0.129- 0.370	-1.523	0.000	0.218	0.128–0.370	-1.523	0.000	0.218	0.129–0.370
	Above 2birth	-2.391	0.000	0.092	0.054–0.155	-2.391	0.000	0.092	0.054–0.155	-2.391	0.000	0.092	0.054–0.155
Occupation (Ref:notworking)	Agricultural	0.171	0.475	1.187	0.742–1.899	0.1712	0.475	1.187	0.742–1.899	0.1712	0.475	1.187	0.742–1.899
	Non Agricultural	-0.089	0.689	0.915	0.591–1.415	-0.0891	0.689	0.915	0.591–1.415	-0.0891	0.689	0.915	0.591–1.415
Wealth Index (Ref:poorest)	Poorer	1.003	0.133	2.727	0.735–10.11	-0.2648	0.375	0.767	0.428–1.378	0.2438	0.397	1.276	0.726- 2.242
	Middle	0.232	0.421	1.261	0.717–2.220	0.2321	0.421	1.261	0.717–2.219	0.2321	0.421	1.261	0.717- 2.220
	Richer	0.0551	0.853	1.057	0.590–1.892	0.0551	0.853	1.057	0.590–1.892	0.0551	0.853	1.057	0.590–1.892
	Richest	0.7331	0.033	2.08	1.061–4.084	0.7331	0.033	2.08	1.061–4.084	0.7331	0.033	2.08	1.061–4.084
Smoking (Ref:No)	Yes	-0.4244	0.551	0.654	0.162–2.638	-0.4244	0.551	0.654	0.162–2.638	-0.4244	0.551	0.654	0.162–2.638
Antenatal Visits (Ref:no)	1–3	0.1477	0.458	1.160	0.785–1.712	0.1477	0.458	1.159	0.784- 1.711	0.1477	0.458	1.159	0.785- 1.712
	Above 4	0.4582	0.036	1.581	1.029 -2.429	0.4582	0.036	1.581	1.029- 2.429	0.4582	0.036	1.581	1.029–2.429
Marital sta (Ref:unmarried)	Married	0.4181	0.369	1.519	0.610–3.783	0.4181	0.369	1.519	0.610–3.783	0.4181	0.369	1.519	0.610–3.783
Visit health Facility in 12 months	Yes	0.3998	0.022	1.49	1.059–2.102	0.3998	0.022	1.49	1.059–2.102	0.3998	0.022	1.49	1.059–2.102
Religion (Ref:orthodox)	Muslim	0.084	0.792	1.088	0.582–2.032	0.084	0.792	1.088	0.582–2.032	0.084	0.792	1.088	0.582–2.032
	Others	0.064	0.849	1.066	0.553–2.053	0.064	0.849	1.066	0.553–2.053	0.064	0.849	1.066	0.553–2.053
	-cons	8.8723	0.000			4.6828	0.000			4.188	0.000		

*Key: LR chi2 (48)* = *793.78 Prob* > *chi2* = *0.000 pseudo R2* = *0.3746*

The variable’s region (Oromia), residency, and parity (above 6) violated the parallel lines’ assumption in the partial proportional odds model. The model, therefore, allows the coefficients of these variables to vary across the response categories. From the PPOM results, region, iron taking status, number of births in last five years, wealth index; the number of antenatal care visits during pregnancy, visit health facility in last 12 months, residence type, educational level, and parity were significantly related with anemia status of pregnant women.

### Predictors that violate the parallel line assumption

PPOM data showed that, when all other variables were held constant, a pregnant woman in Oromia was roughly 76% (OR = 0.242, *p*-value = 0.030) less likely to be moderate, mild, or not anemic than a pregnant woman in Tigray. The fitted model revealed that pregnant women in rural locations were roughly 90% (OR = 0.1, *p*-value = 0.021) less likely to be moderate, mild, or not anemic than pregnant women in urban areas. Similarly, as compared to pregnant women from urban areas, pregnant women from rural areas were roughly 60% (OR = 0.402, *p*-value = 0.003) and 79% (OR = 0.211, *p*-value = 0.000) less likely to be moderate or not anemic and not anemic, respectively.

The result of this study revealed that as compared to pregnant women having no children, pregnant women having six or more children were around 86% (OR = 0.137, *p*-value = 0.000) less likely to be moderate, mild, and not anemic rather than severe anemic. Similarly, compared to pregnant women who had no children, pregnant women who had six or more children were roughly 88% (OR = 0.122, *p*-value = 0.000) less likely to be mild or not anemic.

### Predictors that do not violate the parallel line assumption

Holding all other variables constant, a pregnant woman in Afar was more likely to report worth anemia status than pregnant women in Tigray (OR = 0.447, p-value = 0.004), according to the results of PPOM. A pregnant lady in Somali was also less likely to report better anemia status than a pregnant woman in Tigray (OR = 0.209, p-value 0.001). When compared to pregnant women in Tigray, pregnant women in Dire Dawa were roughly 77% (OR = 0.231, p-value 0.011) less likely to report better anemia status. Holding all other variables constant, compared with pregnant women who do not take iron, pregnant women who take iron were around 3.7 (OR = 3.685 CI = 2.407–5.642) times more likely to report better anemia status. Holding other variables constant, pregnant women having one birth in the last five years tend to report worse anemia status than pregnant women with no birth in the last five years (OR = 0.218, *p*-value = 0.000). Similarly, compared with a pregnant woman who have no birth in the last five years, pregnant women who have had two or more births in the last five years tend to be more likely to report worse anemia status (OR = 0.092, *p*-value = 0.000).

The fitted model showed that compared with the poorest pregnant women, the richest pregnant women were 2.1 (OR = 2.08, *p*-value = 0.033) times more likely to report better anemia status. In comparison to pregnant women who do not visit ANC during pregnancy, the fitted model had shown that pregnant women who visit ANC more than three times during pregnancy were 1.6 (OR = 1.581, *p*-value = 0.036) times more likely to report better anemia status. Keeping all other variables constant, pregnant women who visit health facilities in the last 12 months were 1.5 (OR = 1.49, *p*-value = 0.022) times more likely to be in moderate, mild, or not anemic than pregnant women who do not visit health facilities in the last 12 months. Similarly, pregnant women who have visited a health facility in the last 12 months were 1.5 (OR = 1.49, *p*-value = 0.022) times more likely to be mild or not anemic as compared to pregnant women who have not visited a health facility in the last 12 months.

### Marginal effects

The average marginal effect result (Table 4) revealed a significant marginal effect for region (Afar, Somali, and Dire Dawa), educational level, iron taking status, residence, parity, number of births in the last five years, ANC visit more than three times, and visit health facility in the last 12 months.

**Table 4:** Average marginal probability effects (AMPE) of predictors on anemia status, EDHS 2016 (*n* = *1053*)

Predictors		Severe		Moderate		Mild		Non anemic	
		MPE1	*P*-value	MPE2	*P*-value	MPE3	*P*-value	MPE4	*P*-value
Region	Afar	0.0133	0.015	0.0678	0.000	0.0102	0.020	-0.0913	0.038
	Amhara	-0.0031	0.539	-0.0224	0.526	-0.0064	0.531	0.0319	0.526
	Oromia	0.031	0.079	-0.0544	0.155	0.0259	0.390	-0.0025	0.961
	Somali	0.0366	0.001	0.135	0.001	0.0109	0.000	-0.1825	0.001
	Benishangul	0.0032	0.607	0.0200	0.609	0.0043	0.627	-0.0275	0.610
	SNNPE	-0.0036	0.473	-0.0273	0.440	-0.008	0.421	0.0393	0.436
	Gambela	0.0054	0.479	0.0322	0.467	0.0063	0.486	-0.0439	0.466
	Harari	0.117	0.157	0.0616	0.154	-0.095	0.278	-0.0836	0.157
	AddisAbaba	0.0105	0.345	0.056	0.273	0.0092	0.293	-0.076	0.271
	Dire Dawa	0.0320	0.037	0.125	0.010	0.0112	0.004	-0.168	0.010
Educational Level	Primary	.0048	0.753	0.013	0.645	-0.014	0.000	0.0316	0.000
	Secondary	-0.0346	0.000	-0.1236	0.000	-0.081	0.009	0.0773	0.012
	Higher	-0.0262	0.000	-0.1114	0.000	-0.0254	0.020	0.1630	0.000
Iron take	Yes	-0.0234	0.000	-0.093	0.000	-0.026	0.001	0.143	0.000
Residence	Rural	0.0345	0.000	0.0486	0.059	0.0832	0.000	-0.1663	0.000
Parity (TNCEB)	1–2	0.0082	0.034	0.0503	0.009	0.03	0.020	-0.0885	0.010
	3–5	0.0159	0.004	0.0846	0.000	0.044	0.001	-0.1445	0.000
	Above 6	0.0401	0.000	0.1695	0.000	0.2165	0.000	-0.4261	0.000
Births in last 5 years	1 Birth	0.0151	0.000	0.1008	0.000	0.054	0.000	-0.1699	0.000
	Above2birth	0.0376	0.000	0.1800	0.000	0.0682	0.000	-0.2858	0.000
Wealth Index	Poorer	-0.0203	0.053	0.0450	0.097	-0.0500	0.045	0.0253	0.397
	Middle	-0.0062	0.402	-0.0144	0.428	-0.0036	0.444	0.0241	0.421
	Richer	-0.0015	0.851	-0.0034	0.854	-0.0008	0.854	0.0058	0.853
	Richest	0.0266	0.074	-0.045	0.021	-0.0075	0.064	0.0789	0.029
Antenatal Visits	1–3	-0.0039	0.451	-0.0104	0.461	-0.002	0.482	0.0164	0.458
	Above 4	-0.011	0.029	-0.0322	0.041	-0.006	0.101	0.049	0.037
Visit health Facility in last12month	Yes	-0.0107	0.032	-0.0268	0.020	0.0044	0.070	0.0419	0.020

The fitted AME revealed that as a region shifts from Tigray to Somali and Dire Dawa, the likelihood of pregnant women in Somali and Dire Dawa being non-anemic decreases by 18 (AME = -0.1825, *p*-value = 0.001) and 17 (AME = -0.168, *p*-value = 0.01) percentage points, respectively.

When comparing pregnant women in Afar to pregnant women in Tigray, the probability of being non-anemic drops by about 9 (AME = -0.0913, *p*-value = 0.038) percentage points.

Pregnant women's chances of being moderately anemic increased by 13.5 (AME = 0.135, *p*-value = 0.001) and 12.5 (AME = 0.125, *p*-value = 0.010) percentage points as we went from Tigray to Somalia and Dire Dawa, respectively.

The result of AME showed that the probability of secondary and higher educated pregnant women’s to be moderate anemic would decrease by approximately 12 (AME = -0.1236, *p*-value = 0.00) and 11 (AME = -0.1114, *p*-value = 0.000) percentage points respectively as compared to uneducated pregnant women. Similarly, as compared to uneducated pregnant women, higher educated pregnant women’s probability of being non-anemic would increase by approximately 16 (0.163, *p*-value = 0.000) percentage points. When compared to pregnant women who do not take iron pills, the probability of being severely anemic drops by about 2 (AME = -0.0234, *p*-value = 0.000) percentage points, whereas the probability of being non-anemic rises by about 14 (AME = 0.143, *p*-value = 0.000) percentage points.

Based on the fitted AME model, as residence changes from urban to rural, rural pregnant women’s probability of being non-anemic would fall by approximately 17 (AME = -0.1663, *p*-value = 0.000) percentage points. As compared to pregnant women who have no child, Pregnant women’s probability who have six or more children be severe, moderate and mild anemic would increase by approximately 4(AME = 0.0401, *p*-value = 0.000), 17 (AME = 0.1695, p-value = 0.000) and 22 (AME = 0.2165, *p*-value = 0.000) percentage points respectively whereas the probability of pregnant women be non-anemic would fall by approximately 43 (AME = -0.4261, *p*-value = 0.000) percentage points.

Holding all other variables constant, the likelihood of pregnant women having one birth and two or more births in the last five years being non-anemic would decrease by 17 (AME = -0.1699, *p*-value – 0.000) and 29 (AME = -0.2858, *p*-value = 0.000) percentage points, respectively, compared to pregnant women who have had no births in the last five years. Pregnant women who frequent health facilities in the last 12 months have a lower risk of becoming seriously anemic by about 1(AME = -0.0107, *p*-value = 0.000) percentage points than pregnant women who do not visit health facilities in the last 12 months.

## Discussion

The anemia status of pregnant women was assessed and classified as an ordinal outcome in this study based on blood hemoglobin concentration levels. Anemia was found to be present in 37.51% of pregnant women, with 3.04% being extremely anemic, 17.28% moderately anemic and 17.19% mildly anemic. The data were fitted with partial proportional odds, generalized ordered logit, adjacent category logit, and continuation ratio models, and model comparisons were done. Thus, PPOM is the best fit based on AIC and BIC values, and it was used to identify important drivers of anemia status in pregnant women, with PPOM parameter estimates reported and explained for the significant predictors (at a 5% significance level). The pregnant woman's region, iron taking status, number of births in the last five years, wealth index, number of antenatal care visits, health facility visits in the last 12 months, residence, educational level, and parity are all significant factors related with anemia status.

The study discovered that the pregnant woman's anemia status was greatly influenced by her geographic location. This finding is in line with research from Ethiopia [27–29] which found that the risk of anemia was higher in the Afar, Somali, and Dire Dawa regions. When compared to pregnant women in urban regions, pregnant women from rural areas were more likely to be in the worst category of anemia status. This finding is consistent with research conducted in Ethiopia [30–32] and Tanzania [33]. The reason for this could be a lack of health-care facilities, poor health-seeking behavior, or insufficient basic infrastructure, so concerned bodies are better to act accordingly to eradicate these problems.

The study also discovered that the level of education of pregnant women is a potential indicator of anemia status. A woman with primary, secondary or higher education had a lower likelihood of having a worse anemic status than a woman with no education. This conclusion is supported by research from Ethiopia [13, 30], Eastern Africa [34], and Pakistan [35]. This could be because educated moms have a better understanding of nutrition and eat a wider variety of meals, resulting in a decrease in nutritional deficiency anemia and it is better to encourage and facilitate women education such a problem. Pregnant women with the highest economic status had the lowest risk of anemia when compared to those with the lowest economic status, according to findings from Ethiopian privies research. Studies in poor nations such as Ethiopia[27] and sub-Saharan Africa [36] back with this conclusion. This could be due to the fact that having a low salary means having less money to buy iron-rich foods or eat a well-balanced diet.

This study's findings suggest that not only ANC visits during pregnancy, but also trips to health facilities in the preceding twelve months, can help pregnant women to minimize their anemia burden. This research is in line with previous research in southern Ethiopia [37], Tanzania [38], and sub-Saharan Africa [36]. Pregnant women who attended ANC follow-up were encouraged to eat iron-rich foods and take iron-folic acid pills by health professionals, and prenatal care counseling can assist pregnant women remember to take iron-rich foods and iron-folic acid tablets.

Another finding of this study indicated that pregnant women who took iron were decreased in the risk of anemia. This result is consistent with the past studies [39–42]. The likely explanation for this link is that women require greater iron supplementation during pregnancy, which prevents anemia and also aids in the formation and oxygenation of blood cells, lowering the risk of anemia [37], so the responsible bodies are better to advice the women to take iron during pregnancy.

Findings of this study showed that the higher the total number of children ever born, the higher the risk of anemia for pregnant women. This result agreed with studies in Ethiopia [37, 43] and East Africa [34]. This could be because having more children could lead to food instability in the home and women eating an imbalanced diet.

The study also addressed the individual explanatory variables' marginal effect on a single level of anemia. As a result, the categories Somali, Dire Dawa, higher education, iron intake, rural residence, and a total number of children ever born above six were highly affected by a single response of anemia.

## Strength and limitation of the study

The strength could be the high response rate, and the study was based on numerous variables by considering the ordinal property of anemia status. The study also indicates the marginality of each explanatory variable.

Because the data was obtained before five years, the EDHS 2016 data utilized in this study may not reflect the current situation of anemia status of pregnant women in Ethiopia. Due to numerous missing values, numerous crucial explanatory variables such as vitamin A, gestational age, and HIV status of pregnant women were omitted from this study. Furthermore, because the EDHS were a questionnaire-based survey that relied on the respondents' memories, recall bias in the data could be a flaw in this study.

## Conclusions

Anemia was present in 37.51% of pregnant women, according to our findings. According to the results of the fitted partial proportional odds model, pregnant women's region, residence, number of antenatal care visits, parity, and iron taking status, number of births in the previous five years, educational level, wealth index, and health facility visit in the previous 12 months were all found to be significantly associated with anemia status. Nutrition counseling and education should aim to raise awareness of the consumption of iron-rich foods and participation in antenatal care, and to promote family planning.

Multifactorial therapies are indicated to ameliorate the anemia status of pregnant women. Policy interventions aimed at reducing to lower the risk of anemia are best could be implemented to improve access to health care by providing basic services. Further research should look at multilevel analysis to deal with the hierarchical nature of the data and reduce regional discrepancies in the prevalence of anemia in pregnant women in Ethiopia.

## Data Availability

The datasets used and /or analyzed during the current study available from the corresponding author on reasonable request.

## Abbreviations

PPOM: Partial proportional odds model
GOLM: Generalized ordered logit model
LRT: Likelihood ratio test
MLE: Maximum likelihood estimate
AIC: Akaike information criteria
EDHS: Ethiopian demographic health survey
EA: Enumeration area
ME: Marginal Effect
WHO: World health organization
HICS: Health care incident command
ANC: Antenatal care
LMIC: Low and medium income countries
FMoH: Federal ministry of health
EPHI: Ethiopian public health institute
